# Associations between healthy eating patterns and indicators of metabolic risk in postmenopausal women

**DOI:** 10.1186/1475-2891-9-64

**Published:** 2010-12-08

**Authors:** Ana P Tardivo, Jorge Nahas-Neto, Eliana AP Nahas, Nailza Maesta, Marcio AH Rodrigues, Fabio L Orsatti

**Affiliations:** 1 Department of Gynecology and Obstetrics, Botucatu Medical School, UNESP- Sao Paulo State University, Brazil; 2 Nutrition and Exercise Metabolism Center of Department of Public Health, Botucatu Medical School, UNESP- Sao Paulo State University, Brazil

## Abstract

**Background:**

Since human diets contain many components that may work synergistically to prevent or promote disease, assessing diet quality may be informative. The purpose of this study was to investigate the association between quality diet, by using Healthy Eating Index (HEI), and metabolic risk indicators in postmenopausal women.

**Methods:**

This cross-sectional study included a total of 173 Brazilian women, aged 45-75 years, seeking healthcare at a public outpatient center. Food consumption assessed by 24 h-recall food inquiry was used to calculate HEI scores: >80 implied diet good, 80-51 diet "needed improvement", and <51 diet poor. Anthropometric data included: body mass index (BMI = weight/height^2^), waist-circumference (WC), body fat (%BF) and lean mass (%LM). Data on total cholesterol (TC), high density lipoprotein cholesterol (HDLC), low density lipoprotein cholesterol (LDLC), and triglycerides (TG) were also collected. Fisher's Exact test, and logistic regression method (to determine odds ratio, OR) were used in the statistical analysis.

**Results:**

Overweight and obesity were observed in 75.7% of the participants. Excessive %BF (> 35%) was observed in 56.1%, while %LM was reduced (< 70%) in 78.1%. WC was elevated (≥88 cm) in 72.3%. Based on HEI values, diet quality was good in 3% (5/173), needed improvement in 48.5% (84/173), and was poor in 48.5% (84/173) of the cases. In this group, 75% of women had high intakes of lipids (> 35%), predominantly saturated and monounsaturated fat. On average, plasma TC, LDLC, and TG levels were higher than recommended in 57.2%, 79.2% and 45.1% of the women, respectively, while HDLC was low in 50.8%. There was association between HEI scores and the %BF that it was higher among women with HEI score < 80 (p = 0.021). There were not observed significant risk associations between HEI and lipid profile.

**Conclusion:**

Among the Brazilian postmenopausal women attending a public outpatient clinic, diet was considered to need improvement or to be of poor quality, attributed to high saturated fat ingestion, which probably caused a negative impact on metabolic risk indicators, namely body composition.

## Background

Menopause is frequently associated with weight gain and a shift in body fat distribution. Once estrogen deficiency is established, a new pattern of fat distribution is observed - gluteo-femoral or gynecoid fat deposition decreases and abdominal or android fat accumulation increases [1,2]. Abdominal obesity is metabolically different from gynecoid obesity and contributes for the development of insulin-resistance, type 2 diabetes mellitus, and dyslipidemia, components of metabolic syndrome, which are important risk markers of cardiovascular disease (CVD), the major cause of death among postmenopausal women [3]. Metabolic syndrome (MetS) is highly prevalent (39.6%) among Brazilian postmenopausal women seeking gynecologic care, and abdominal obesity has been reported to be a strong MetS predictor [4]. Moreover, behavioral factors such as lifestyle, diet, sedentarism, smoking and alcohol use are associated with the onset and progress of atherosclerotic disease [5,6].

Diet and eating habits have a pivotal role in maintaining human health. Unhealthy eating, obesity and nutritional deficiencies may lead to various diseases [7]. Modern dietary patterns, characterized by higher energy density, predispose to non-transmittable diseases such as type 2 diabetes mellitus, arterial hypertension and coronary heart disease that are directly associated with overweight and obesity [8,9]. Weight gain is related to the energy imbalance in which energy intake is greater than energy expenditure. This imbalance is influenced by diet quality, nutrient body metabolism and sedentarism associated with genetic susceptibility [10]. Thus, in order to treat and prevent diseases highly prevalent among postmenopausal women, the nutritional assessment of macronutrient (proteins, glycids and lipids) and micronutrient (calcium and iron) intake is necessary [11].

Since human diets contain many components that may work synergistically to prevent or promote disease, assessing diet quality may be more informative [12]. Indexes of dietary quality have been developed in recent years to address this shortcoming in nutrition research. The Healthy Eating Index (HEI) was developed to measure adherence to dietary guidelines (Food Guide Pyramid) and was shown to adequately measure overall diet quality [13]. Poor diet is a risk factor for many chronic diseases, including CVD. HEI is a validated instrument, that has been employed in several studies correlating the quality of diet and chronic diseases [12,14,15]. Relatively poor dietary quality, characterized by higher composite nutritional risk (higher dietary lipids and lower intakes of fiber and micronutrients), may predict the development of abdominal obesity independent of age, physical activity, or menopausal status [14].

Within this context, the primary purpose of this study was to investigate the association between dietary quality, by using a validated measure (HEI), and metabolic risk indicators in Brazilian postmenopausal women.

## Methods

### Participant selection and study design

This cross-sectional study was undertaken using a convenience sample consisting of 389 Brazilian postmenopausal women attending healthcare at a public outpatient center in Southeastern Brazil, between January and December 2009. All women aged ≥ 45 years, without menstruation for at least 12 months, and in good health (self-rated) that agreed to participate were included in the study. A total of 216 women were excluded due to: (1) incomplete data; (2) refusal to undergo planned assessment procedures; (3) extremely low or high reported energy intakes (< 500 or > 4,000 Kcal/d); (4) ongoing dietary counseling with a doctor or dietitian; (5) use of drugs known to alter lipid metabolism; (6) non-controlled diabetes or thyroid diseases; and (7) special or vegetarian diet. All subjects were from low socioeconomic groups (income ≤ US$ 500 per month). The study population was homogeneous and representative of the population attending in our service. Informed consent was obtained from all subjects and the Research Ethics Committee of Botucatu Medical School, Sao Paulo State University/UNESP, approved the study.

### Dietary intake measurements

Initial evaluation consisted of a health questionnaire and general physical examination. Data collected included information on age, time since menopause, parity, hormone therapy (HT) use, physical activity and history of high-blood pressure, diabetes, cardiovascular disease, obesity, osteoporosis, and dyslipidemia. Women doing regular physical exercises for 30 minutes of moderate-intensity aerobic activity at least five times a week or muscle-strengthening activities on two or more days a week were considered to be active [16].

A 24-hour dietary recall was obtained from every participant by a trained dietary interviewer (Tardivo, AP). The stepwise method, adapted from Thompson and Byers, was used [17]. The type and amount of foods consumed were recalled using recall aids such as abstract food models, special charts, measuring cups, and rulers to help in quantifying the amounts consumed. Special probes were used to help the recall of commonly forgotten items such as condiments, accompaniments, fast foods, etc. The nutritive value of the foods was also checked, especially with regard to the nutrients that are considered to be the greatest source of nutritive value for these foods. Thus, the 24-hour dietary recall data were controlled for errors that might have occurred in estimating nutrient intake. Based on these data, dietary habits as well as the dietary intake of proteins, carbohydrates, fat, and all known essential micronutrients were quantified using the Nutrition Software ("NutWin") [18].

Dietary intake data, as assessed by the 24-h recall, were used to calculate Healthy Eating Index (HEI) scores. HEI comprises 10 components (saturated fat, total fat, cholesterol, sodium, grain, fruit, dairy, meat, and vegetable intakes, plus a measure of dietary variety), each contributing 10 points to the maximum possible score of 100 [13]. Food serving amounts were computed from food consumption data using factors derived from the serving-size assumptions given in the Food Guide Pyramid [19]. Ten points were scored if saturated fat ≤ 10% of energy, total fat ≤ 30% of energy, cholesterol ≤ 300 mg, and sodium ≤ 2.4 mg. A zero score reflected ≥ 15% of energy for saturated fat, ≥ 45% of energy for total fat, cholesterol ≥ 450 mg, and sodium ≥ 4.8 mg; between these two cutoff points, scores were scaled proportionately. A similar process was used for food groups (grains, fruits, vegetables, dairy, and meat): consumption of the recommended number of servings for the individual's age and sex resulted in a score of 10, with a score of 0 if no servings were consumed [20]. The maximum overall score for the 10 components combined is 100. High component scores indicated intakes close to recommended ranges or amounts; low component scores indicated lower compliance. HEI scores > 80 implied diet was "good", between 51 and 80 implied diet "needed improvement", and < 51 implied diet was "poor" [21].

### Anthropometric measurements

Anthropometric data included body mass index (BMI = weight/height^2^), waist circumference (WC), waist/hip ratio (WHR), percentage of body fat (%BF) and percentage of lean mass (%LM). All anthropometric measures were taken under fasting conditions, and participants were wearing light-weight clothing and no shoes. The body mass index (BMI = weight/height^2^) was used to assess weight variation. Height and weight were determined with a stadiometer (Seca^®^, Brazil) and a standard balance beam scale (Filizola^®^, Brasil), respectively. Weight was classified according to the system used by the World Health Organization (2002): 18.5 -24.9 kg/m^2 ^= normal weight; 25- 29.9 kg/m^2 ^= overweight; ≥ 30.0 kg/m^2 ^= obesity.

Waist circumference (WC) was measured to the nearest 0.5 cm midway between the lowest rib margin and the top of iliac crest, and hip circumference at the largest posterior extension of the buttocks. Both were measured to the nearest 1 cm with an inelastic tape. Measurements were taken at the end of a normal respiration while subjects stood erect with arms hanging loosely at sides and feet were together. Abdominal fat was indirectly assessed by measuring waist circumference and was considered high when waist >88 cm [22]. The waist/hip ratio (WHR) was used to assess body fat distribution considering WHR < 0.80 as a gynecoid pattern and WHR ≥ 0.80 as an android pattern.

Body fat density was determined by the equation of Jackson et al. [23] based on tricipital, supra-iliac and thigh skin fold measurements that were taken three times with a Lange Skin fold Caliper (0.1-mm precision). Once density was calculated, the equation of Siri [24] was used to estimate body fat percentage. Adiposity was considered good when percentage < 25-30% of total body weight. Lean mass (LM) was obtained by subtracting absolute fat (Kg) from total body weight and converting the result into percentage. LM was considered adequate when > 70% [25].

### Laboratory assessment

Blood was collected from each subject after 12-hour fasting. A 12-ml blood sample was drawn via venipuncture into vacuum tubes (Vacutainer^®^, England). Triglycerides (TG), total cholesterol (TC), high density lipoprotein cholesterol (HDLC), low density lipoprotein cholesterol (LDLC), and glucose levels were measured by the automatic biochemical analyzer Vitros 950 (Jonhson & Jonhson, USA). Total cholesterol (TC), HDLC, triglycerides (TG) and glucose levels were quantified by the colorimetric test which provides a linear response over up to 800 mg/dl for triglycerides and 900 mg/dl for total cholesterol. LDLC was calculated using the formula of Friedewald et al. (1972), where total cholesterol is subtracted from the sum of HDLC and triglyceride divided by five. Normality rates were: TC < 200 mg/dl, HDLC > 50 mg/dl, LDLC < 100 mg/dl, TG < 150 mg/dl and glucose < 110 mg/dL [22].

### Statistical analysis

A non-probability purposive sample was used. Sample size was determined based on the number of patients/year attending the Climacterium Outpatient Service of Botucatu Medical School, Sao Paulo State University - UNESP who met study inclusion criteria. Based on the data collected (quantitative variables), tables including patient characteristics and laboratory values expressed as median and interquartile range were generated. The classification variables were shown in numbers and percentage between parentheses. In the association of variables, patients were divided according to the values of HEI: >80, between 80-51 and < 51. For categorical variables, the comparison between groups was performed by Fisher's Exact Test.

Spearman correlation was used to assess correlations of dietary intake quality (HEI) with body composition parameters. Multivariate analysis, using a logistic regression model (odds ratio), was used to evaluate the influence of diet on lipid profile. In the model, the HEI value was considered as the independent variable while total cholesterol, HDLC, LDLC, and triglycerides were considered dependents variables. Age, time of menopause, and body mass index were used as adjusting variables in the models. Variable input to the regression model was considered at a significance level of 20% using a stepwise procedure. The statistical tests were bilateral, and the level of significance adopted was 5%. The statistical analyses were processed by using software SAS, version 9.2 for Windows.

## Results

Clinical characteristics and dietary intakes are shown in Table 1. Median age was 54.0 years and time since menopause was 6.0 years. The analysis of food intake indicators revealed that total daily calorie intake averaged 1,607.8 kcal, corresponding to 27 kcal/kg weight/day. Protein caloric contribution averaged 15.4%; carbohydrates (46.0%) and lipids (38.3%) were the greatest sources of energy. Median saturated, polyunsaturated and monounsaturated fat intake was 8.8%, 10.4% and 13.0%, respectively (Table 1). Cholesterol uptake was considered adequate (< 300 mg) in 88% of the cases. On the other hand, calcium uptake (> 1200 mg) and iron uptake (> 18 mg) reached recommended levels in only 9% and 7% of the subjects, respectively. The median HEI score was 60.0, diet need improvement (Table 1).

**Table 1:** Clinical characteristics, dietary intakes, laboratory values and diet quality score for all subjects (n = 173).

Parameters	Median (25^th^; 75^th^)*	Range**
*Clinical Characteristics*		
Age (years)	54.0 (49.0; 59.5)	45-75
Age at menopause (years)	48.0 (43.0; 51.0)	33-55
Time since menopause (years)	6.0 (2.0; 11.0)	1-33
*Anthropometric measurement*		
BMI (kg/m^2^)	28.3 (25.2; 31.5)	19.1-42.3
Waist circumference (cm)	94.5 (87.1; 102.5)	68-129.5
WHR	0.88 (0.81; 1.0)	0.50-1.21
Body fat (%)	36.3 (31.7; 39.8)	20.5-51.5
Lean Mass (%)	63.7 (60.1; 68.3)	48.5-84.7
*Dietary Intakes*		
Total energy intake (kcal/d)	1607.8 (1260.5; 1907.2)	706.1-3617.8
Proteins (%)	15.4 (12.6; 18.9)	6.9-38.8
Carbohydrate (%)	46.0 (40.1; 54.3)	24.1-66.5
Lipid (%)	38.3 (29.2; 44.3)	18.6-68.4
Saturated fat (%)	8.8 (6.3; 11.7)	3.2-18.2
Monounsaturated fat (%)	10.4 (8.2; 13.0)	4.3-20.2
Polyunsaturated fat (%)	13.0 (9.1; 16.8)	4.6-29.7
HEI total score	60.0 (50.0; 70.0)	30-90
*Biochemical Markers*		
Glucose (mg/dl)	94.0 (88.0; 99.5)	75-216
Total Cholesterol (mg/dL)	210.0 (180.0; 239.5)	123-396
HDLC (mg/dL)	49.0 (40.5; 58.0)	28-92
LDLC (mg/dL)	127.8 (103.5; 156.5)	53-299.8
Triglycerides (mg/dL)	151.0 (89.5; 189.0)	54-361

BMI, body mass index; WHR, waist/hip ratio; %, percentage; HEI, Healthy Eating Index; high density lipoprotein cholesterol, HDLC; low density lipoprotein cholesterol, LDLC.

*Data are expressed as median with 25^th ^and 75^th ^percentiles in parentheses.

** Values minimum - maximum.

Based on HEI values, diet quality was good in 3% (5/173), needed improvement in 48.5% (84/173), and was poor in 48.5% (84/173) of the cases. The associations of descriptive characteristics according to the HEI scores were shown in Table 2. Overweight and obesity were present in 75.7% (131/173) of the participants. Excessive body adiposity (> 35%) was observed in 56.1% (97/173) of the subjects. Most women (72.3%) showed greater fat concentration in the abdominal region (WC > 88 cm) while lean mass was below normal (< 70%) in 78.1%. Of the 173 women assessed 70 (40.4%) were on hormone therapy (HT) (Table 2). None association was observed between HEI scores and clinical variables analyses (p > 0.05). Except for the percentage of body fat that it was higher among women with diet need improvement or poor (p = 0.021) (Table 2). There were no significant correlations between the values of HEI with BMI (r = -0.52, p = 0.495), WC (r = -0.24, p = 0.753), %BF (r = -0.139, p = 0.069), and %LM (r = 0,138, p = 0.71).

**Table 2:** Associations between diet quality scores (Healthy Eating Index, HEI) and clinical characteristics, dietary intakes, and biochemical markers of postmenopausal women (n = 173).

Characteristics	N	Diet Good HEI > 80 (n = 5)	Diet Need Improvement HEI 80-51(n = 84)	Diet poor HEI < 51 (n = 84)	p value*
*Clinical Characteristics*					0.779
Age group (years)					
≤ 49	44	0 (0.0)	23 (52.3)	21 (47.7)	
50-59	86	4 (4.7)	40 (46.5)	42 (48.8)	
≥ 60	43	1 (2.4)	21 (48.8)	21 (48.8)	
Age of menopause (years)					0.933
≤ 40	29	0 (0.0)	15 (51.7)	14 (48.3)	
≥ 41	144	5 (3.5)	69 (47.9)	70 (48.6)	
Menopause duration (years)					0.852
≤ 5	85	3 (3.5)	42 (49.4)	40 (47.1)	
6-10	45	1 (2.2)	19 (42.2)	25 (55.6)	
> 10	43	1 (2.3)	23 (53.5)	19 (44.2)	
Marital Status					0.619
with partner	124	4 (3.2)	57 (46.0)	63 (50.8)	
without partner	49	1 (2.0)	27 (55.1)	21 (42.9)	
BMI (kg/m^2^)					0.757
< 25	42	1 (2.4)	21 (50.0)	20 (47.6)	
25 - 29,9	68	3 (4.4)	35 (51.5)	30 (44.1)	
≥ 30	63	1 (1.6)	28 (44.4)	34 (54.0)	
Waist Circumference (cm)					0.379
< 88	48	0 (0.0)	22 (45.8)	26 (54.2)	
≥ 88	125	5 (4.0)	62 (49.6)	58 (46.4)	
WHR					0.707
< 80	32	0 (0.0)	17 (53.1)	15 (46.9)	
≥ 80	141	5 (3.6)	67 (47.5)	69 (48.9)	
Body fat (%)					**0.021**
< 25	12	1 (8.3)	3 (25.0)	8 (66.7)	
25-35	64	4 (6.2)	25 (39.1)	35 (54.7)	
≥ 35	97	0 (0.0)	56 (57.7)	41 (42.3)	
Lean Mass (%)					0.279
< 70	136	4 (2.9)	70 (51.5)	62 (45.6)	
≥ 70	37	1 (2.7)	14 (37.8)	22 (59.5)	
Use of hormone therapy					0.316
Yes	70	3 (4.3)	40 (57.2)	27 (38.5)	
No	103	2 (1.9)	44 (42.7)	57 (55.4)	
Physical exercise					0.406
Yes	35	0 (0.0)	20 (57.1)	15 (42.9)	
No	138	5 (3.6)	64 (46.4)	69 (50.0)	
Smoking					0.264
Yes	45	0 (0.0)	26 (57.8)	19 (42.2)	
No	128	5 (3.9)	58 (45.3)	65 (50.8)	
High-Blood Pressure (mmHg)					0.801
Yes	70	1 (1.4)	35 (50.0)	34 (48.6)	
No	103	4 (3.9)	49 (47.6)	50 (48.5)	
*Dietary Intakes*					
Total energy intake (kcal/d)					**0.005**
< 2000.0	135	4 (3.0)	60 (44.4)	71 (52.6)	
≥ 2000.0	38	1 (2.6)	24 (61.2)	13 (34.2)	
Proteins (%)					**0.009**
< 10	16	0 (0.0)	5 (31.2)	11 (68.8)	
10-35	153	5 (3.3)	76 (49.7)	72 (47.0)	
> 35	4	0 (0.0)	3 (75.0)	1 (25.0)	
Carbohydrate (%)					**< 0.001**
< 45	79	0 (0.0)	24 (30.4)	55 (69.6)	
45-65	90	5 (5.6)	56 (62.2)	29 (32.2)	
> 65	4	0 (0.0)	4 (100.0)	0 (0.0)	
Lipid (%)					**< 0.001**
< 20	3	0 (0.0)	3 (100.0)	0 (0.0)	
20 - 35	74	5 (6.8)	57 (77.0)	12 (16.2)	
> 35	96	0 (0.0)	24 (25.0)	72 (75.0)	
Saturated fat (%)					**< 0.001**
< 7	54	2 (3.7)	37 (68.5)	15 (27.8)	
≥ 7	119	3 (2.5)	47 (39.5)	69 (58.0)	
Monounsaturated fat (%)					**< 0.001**
< 10	78	4 (5.1)	53 (67.9)	21 (27.0)	
≥ 10	95	1 (1.0)	31 (32.6)	63 (66.4)	
Polyunsaturated fat (%)					**< 0,001**
< 10	59	0 (0.0)	13 (22.0)	46 (78.0)	
10-15	62	2 (3.2)	32 (51.6)	28 (45.2)	
> 15	52	3 (5.7)	39 (75.0)	10 (19.3)	
*Biochemical Markers*					
Glucose (mg/dL)					0.942
≤ 100	132	4 (3.0)	63 (47.7)	65 (49.3)	
> 100	41	1 (2.4)	21 (51.2)	19 (46.4)	
Total Cholesterol (mg/dL)					0.804
< 200	74	3 (4.1)	35 (47.3)	36 (49.6)	
≥ 200	99	2 (2.0)	49 (49.5)	48 (48.5)	
HDLC (mg/dL)					0.884
≥ 50	85	3 (3.5)	40 (47.1)	42 (49.4)	
< 50	88	2 (2.3)	44 (50.0)	42 (47.7)	
LDLC (mg/dL)					0.877
< 100	36	1 (2.8)	16 (44.4)	19 (52.8)	
≥ 100	137	4 (3.0)	68 (49.6)	65 (47.4)	
Triglycerides (mg/dL)					0.573
< 150	95	4 (4.2)	46 (48.4)	45 (47.4)	
≥ 150	78	1 (1.3)	38 (48.7)	39 (50.0)	

Data are expressed in numbers and in percentage between parentheses.

BMI, body mass index; WHR, waist hip ratio; high density lipoprotein cholesterol, HDLC;

low density lipoprotein cholesterol, LDLC.

*Statistical difference between groups p < 0.05 (Fisher's Exact test)

There were significant association between HEI values and dietary intakes in all macronutrients, as noted in Table 2. In the group with diet poor was observed that 75% of women had high intakes (> 35%) of lipids, predominantly saturated and monounsaturated fat (Table 2). On average, plasma total cholesterol, LDLC, and TG levels were higher than recommended in 57.2% (99/173), 79.2% (137/173) and 45.1% (78/173) of the women, respectively, while HDLC was low in 50.8% (88/173). None association was observed between HEI scores and biochemical markers analyses (p > 0.05) (Table 2). In analyzing the impact of diet on lipid profile were not observed significant risk associations between HEI and total cholesterol (OR 0.966; CI 95% 0.51-1.81), HDLC (OR 1.024; CI 95% 0.55-1.91), LDLC (OR 1.194; CI 95% 0.55-2.58), or triglycerides (OR 0.857; CI 95% 0.46-1.59) (date not shown).

## Discussion

The approach to menopause should be multidisciplinary and involve physicians, physical educators, physical therapists and nutritionists to increase survival and quality of life. Aging with quality of life requires appropriate nutritional management involving the balanced intake of macronutrients (carbohydrates, proteins, and lipids) and micronutrients in addition to an increased daily consumption of fruit, vegetables and whole grains (fiber-rich) [26]. Poor diet quality has been considered a major determinant of obesity and is, therefore, an important variable to be investigated, especially in more vulnerable groups such as postmenopausal women [27]. In this study, Healthy Eating Index (HEI) assessment showed that in only 3% of the cases diet was of good quality, while in 48.5% it needed improvement and in 48.5% was of poor quality. Such poor quality was attributed to low whole-grain intake and high saturated fat ingestion, which probably caused a negative impact on metabolic risk indicators, namely body composition and lipoprotein profile.

Several studies have demonstrated the need for instruments capable of assessing dietary intake patterns as a whole in order to avoid the flaws associated with the evaluation of specific nutrients or foods [21,28]. Therefore, it is important to use a method with the ability to measure the quality of the food consumed such as HEI [12,14,15]. In our study, the mean HEI score was 56.6 (diet need improvement), lower than observations for the general US population of women ≥ 50 years (mean HEI score 66.6) [21]. This finding is interesting because it is often assumed that volunteers for clinical trials are more health-conscious than the general population. The overall low diet quality was most likely due to the high intake dietary fat; our participants tended to score low on these variables. These results are in agreement with the study by Boynton et al [15] who found HEI scores (mean 63.1) slightly lower than those in the general US population. Specific data on dietary patterns and metabolic risks in Brazilian postmenopausal women are not available. In a study population of 3454 adults (> 20 years) residing in Sao Paulo State, Brazil, the authors found that only 5% had a good diet, 74% had a diet that needed some degree of improvement and 21% had a poor diet [29]. However, almost 13% of the study sample consisted of subjects aged 60 years or more.

The postmenopausal participants were overweight with increased body fat percentage and waist circumference. Overweight and obesity were present in 39.3% and 36.4%, of the participants, respectively. The results are consistent with data showing that the population is becoming overweight; approximately 66% of the female population in this age group is overweight or obese [30]. Identifying the type of body fat distribution is crucial because the accumulation of fat in the abdominal region is closely related with metabolic changes that can lead to the development of CVD and diabetes mellitus [31]. The Nurses' Health Study demonstrated that a high waist circumference is significantly associated with the increased rate of death by CVD observed in women with normal weight [3]. In our study was observed association between poor quality diet and the percentage of body fat. And the risk for detection of diet poor was significantly higher in women with elevated WC. Similarly to our results, Boynton et al. [15], evaluating 164 postmenopausal women, found that women with higher-quality diets were more likely to have lower percent body fat.

Among study participants, the median of plasma total cholesterol, LDLC and triglycerides were above the desirable levels of the women, and HDLC was low. Besides tending to gain weight, postmenopausal women are susceptible to lipid metabolism changes due to estrogen deficiency that raises total cholesterol and lipoprotein levels producing a lipid profile highly favorable to atherogenesis [32,33]. A diet rich in refined carbohydrates, as observed in this present study, contributes for the development of atherosclerosis and increases cardiovascular risk, especially at 50-69 years of age [22]. Although the association of abnormal lipid profile with individual nutrients and food group has been well established, the relation between diet quality and lipids in women remains undocumented [34]. In this study analyzing the impact of diet on lipid profile were not observed significant risk associations between HEI and lipid profile. This can be attributed to the small number of women (only 3%) with diet good. The vast majority of participants (97.1%) had low-quality of diet, rich in lipids that could reflect the high rate of alteration in lipid profile. In agreement with our results, in the National Health and Nutrition Examination Survey (NHMES III), with 16,467 American adults (> 17 years), lipid profile was not correlated with HEI scores [35].

Several studies investigated the influence of diet on cardiovascular risk by using predefined diet-quality scores [36]. These scores provide an overall estimate of dietary quality by quantifying and summing up a number of nutritional variables (foods and/or nutrients) that are considered to be important to health. In 2009, Hoekstra et al. [37] summarized outcomes of cohort studies on cardiovascular endpoints in women using a range predefined dietary-quality scores. Common components of the dietary-quality scores are intake of fruits and vegetables, cereals and quality of dietary fat. The protective effect of a healthy diet was consistent across all studies with reduction in cardiovascular ranging from 17% to 47% for the high quality versus low-quality diets. Thus, dietary counseling should be an integral part of the cardiovascular risk management in postmenopausal women. Manios et al. [38] studying changes in diet quality score, macro- and micronutrient intake following a nutrition education intervention in 75 postmenopausal women, found that the nutrition education program induced favorable changes for the intervention group in the intake of micronutrients primarily related to bone health and in total fat intake.

The energy intake considered ideal for body weight maintenance in women amounts to 1900 kcal/day and 30 kcal/kg [20]. On average, these were the values obtained by the 24-h recall among study participants who reported the consumption of approximately 1800 kcal and 28 kcal/kg. This information, however, is not consistent with the excessive body adiposity (> 35%) observed in 56.1% of these women. This underestimation of energy intake is likely to be due to the method used, which consists of remembering the amount and type of the food consumed, and the fact that obese individuals tend to underreport the actual amount taken. The assessment of nutrient intake involves comparing the typical daily intake with the requirements of an individual, despite the susceptibility of dietary assessment tools that are inherently prone to error [39]. Previous studies using the 24-h recall method demonstrated that current nutritional status is independent of the food ingested on the day preceding the interview as such dietary intake is too recent to directly influence obesity [40,41].

The Acceptable Macronutrients Distribution Ranges (AMDR) proposed in 2005 by the National Academy of Sciences was 45%-65% for carbohydrates, 10%-35% for proteins, and 20%-35% for lipids. Regarding lipid intake, the ingestion of saturated, polyunsaturated and monounsaturated fat should be < 7%, < 10% and > superior 10-15% [20]. From the quantitative standpoint, the consumption of calories from carbohydrates and proteins observed in this study was within the recommended range. On the other hand, the ingestion of lipids was excessive and qualitatively inappropriate as saturated and polyunsaturated lipids were above recommended levels while monounsaturated lipids were at the lower limit of normality. Such inadequate lipid quality did not contribute for a greater cholesterol intake, which is explained by the type of food consumed.

The findings herein reported should be interpreted with caution as this study had some limitations. First, the sample size was relatively small due to the nature of the study design used (cross-sectional study based on a convenience sample) and the elevated rate of refusals to participate. The study population does not reflect the general population, so the results may not be extended to other population. Second, although 24-hour recalls are frequently used in dietary assessment, intake on a single day is a poor estimator of long-term usual intake; which may under or over estimated the HEI. Third, all subjects were from low socio-economic groups (income ≤ US$ 500 per month) that have no access to nutritional education. Eating habits can be influenced by purchasing power, advertising and practicality in consumption. The rising trend toward highly energy-dense diets is stimulated by food industries that produce tasty foods containing high saturated fat and glucose levels and reduced fiber content at a relatively low cost [8]. This "modern" dietary pattern predisposes to obesity and cardiovascular diseases. Kant & Graubard [42], using data from the National Health and Nutrition Examination Surveys (NHANES) I (1971-1975), II (1976-1980), III (1988-1994) and 1999-2002, examined the independent associations of poverty income ratio (PIR) and education with diet and biomarkers of diet and disease in 25-74-year-olds (n = 36,600). A large PIR differential in the likelihood of reporting a fruit or all five-food groups intake, and an education differential in likelihood of obesity and carbohydrate intake, was noted. Unfavorable dietary and biomarker profiles in Americans with low income and education suggest continued need for improvement in the quality of diets of these high-risk groups.

## Conclusions

Among the Brazilian postmenopausal women attending a public outpatient clinic, diet was considered to need improvement or to be of poor quality, attributed to high saturated fat ingestion, which probably caused a negative impact on metabolic risk indicators, namely body composition.

## Competing interests

The authors declare that they have no competing interests.

## Authors' contributions

JNN, EAPN, NM contributed to the conceptualization and design, interpretation and writing of the article. APT developed the research protocol and conducted the data collection. MAHR and FLO were responsible for data analyses. All authors critically reviewed the manuscript and approved the final version submitted for publication.
